# 

**Published:** 1859-04

**Authors:** 


### Contents of the Chicago Medical Journal, April, 1859.

ORIGINAL COMMUNICATIONS.

PAGE.
ART. I. On the Treatment of Chronic Hydrocephalus. By Daniel Brainard, M.D., Professor of Surgery in Rush Medical College.......... .............. 197
ART. II. Cases illustrating the Practical Use of the Opthalmoscope, as a means of Diagnosis in certain Diseases of the Eye. By E. L. Holmes, M.D., of Chicago, Ill.......................................................... 217
ART. III. Case of Uterine Polypus. By Dr. James Cody, of Watertown, Wis................ ...................... ......... ............................ 220
ART. IV. Marshall Hall's Ready Method. By George V. Ewing, M.D., of Rock Run, Illinois.............................................................. 224

TRANSLATIONS FROM FOREIGN JOURNALS.

Researches on the Possibility of Temporarily Reanimating Persons dying of Disease. By Dr. E. Brown-Sequard. Translated by Thomas Bevan, M.D., from the Journal de Physiologie for October, 1858...................... 227
Researches on the Erectile Organs of the Female. By M. Charles Rouget. Translated by Dr. Bevan..................................... ..................... 234
Circulation of the Nervous Fluid. From the Gazette des Hopitaux. Translated by the Editor.......................... ........................................ 235

BOOK AND PAMPHLET NOTICES.

Course of Comparative Physiology. Given at the Museum of Natural History. By M. Flourens. Paris, 1856............................ ............. 236
On Poisons, in relation to Medical Jurisprudence and Medicine. By Alfred S. Taylor, M.D., F.R.S. Philadelphia: Blanchard & Lea, 1859. Pages 755................................................................................. 241

EXTRACTS.

On Scarlatina and its Treatment. By E. Bishop, M.D........................ 242
On the Speculum Vaginæ. By John P. Mettauer, M.D., L.L.D., of Virginia....................................................... ......................... 246

EDITORIAL.

American Medical Association.............................................. ....... ...

MISCELLANEOUS.

Chicago Academy of the Medical Sciences.................. ............... .. .... 255
Statistics of Medical Colleges.............................................. ................. 256
University of the Pacific.................................................. .................... 257
Titles of Articles in Exchanges.......... ........................ ...................... 258
Chicago Medical Society.................................................................. 260

